# A rare form of calcinosis in patients with systemic sclerosis–myositis overlap: report of four cases

**DOI:** 10.1093/rap/rkae011

**Published:** 2024-01-27

**Authors:** Sheilla Achieng, Jonathan Harris, Muditha Samaranayaka, Ariane L Herrick

**Affiliations:** School of Biological Sciences, Division of Musculoskeletal and Dermatological Sciences, The University of Manchester, Manchester Academic Health Science Centre, Manchester, UK; Department of Rheumatology, Northern Care Alliance NHS Foundation Trust, Salford Care Organisation, Manchester, UK; Department of Radiology, Northern Care Alliance NHS Foundation Trust, Salford Care Organisation, Manchester, UK; Department of Rheumatology, Northern Care Alliance NHS Foundation Trust, Salford Care Organisation, Manchester, UK; School of Biological Sciences, Division of Musculoskeletal and Dermatological Sciences, The University of Manchester, Manchester Academic Health Science Centre, Manchester, UK; Department of Rheumatology, Northern Care Alliance NHS Foundation Trust, Salford Care Organisation, Manchester, UK

**Keywords:** calcinosis, myositis, systemic sclerosis, scleroderma

## Abstract

**Objectives:**

Calcinosis is a well-described entity that occurs in patients with systemic sclerosis (SSc) and dermatomyositis (DM). Calcinosis in SSc typically occurs over pressure points and is usually nodular. We present a case series of four patients with SSc with a much rarer, diffuse form of calcinosis to illustrate this poorly recognized pattern of extensive and debilitating disease.

**Methods:**

Four patients with SSc and extensive calcinosis were identified from patients attending a tertiary rheumatology centre in the preceding 3 years. Their electronic case notes, radiographic images and medical photographs were reviewed.

**Results:**

All four patients had the diffuse cutaneous subtype of SSc (dcSSc) and additionally a myositis overlap. This was in the context of 102 of 461 (22%) patients with SSc whose clinical details had been recorded in the preceding 3 years having dcSSc. Their ages at diagnosis ranged from 27 to 65 years. Three were female, two were anti-Scl70 antibody positive, and two were anti-PMScl antibody positive. Development of calcinosis occurred between 1 and 6 years after onset of SSc. Plain radiography showed very extensive calcinosis in various sites, distributed in a pattern akin to sheets of calcium-containing deposits in the skin and subcutaneous tissue.

**Conclusions:**

Although calcinosis is common in SSc, extensive sheet-like calcinosis is very rare. Our experience suggests that when this form of calcinosis does occur, this is in the context of the diffuse cutaneous subtype of disease and with myositis overlap. The four cases described should raise awareness of this unusual and extensive pattern of disease.

Key messages

- Extensive sheet-like calcinosis is a rare and debilitating form of SSc-related calcinosis.
- This type of calcinosis tends to occur in the context of dcSSc and myositis overlap.
- Further research is needed to identify effective targeted therapies for this rare phenotype of calcinosis.

## Introduction

Calcinosis cutis is the deposition of calcium-containing salts in the subcutanoeus and intracutaneous tissues. It is commonly seen in patients with systemic sclerosis (SSc) and dermatomyositis (DM), although it can also occur in other autoimmune rheumatic diseases, such as systemic lupus erythematosus (SLE). It has a prevalence of 20–40% in patients with SSc [1, 2]. Calcinosis is often debilitating and painful, and it can result in complications, such as skin ulceration and infection [3]. Typically, it develops as nodules over pressure points, for example in the fingers and over extensor surfaces of knees and elbows. In a small number of patients, nodular lesions become very large (pseudotumoral calcinosis) [4]. Very rarely, calcinosis can present in an extensive linear pattern akin to diffuse sheets of calcified deposits, which are not limited to the pressure points. We present a case series of four patients with this extensive pattern of disease, to highlight its clinical burden. We then discuss the challenges in management of calcinosis and briefly discuss possible novel therapies.

## Methods

Four patients with SSc and extensive calcinosis were identified retrospectively from patients attending a tertiary rheumatology centre in the preceding 3 years. Their electronic case notes, radiographic images and medical photographs were reviewed. All patients provided written informed consent. Autoantibody testing in our centre was carried out using multiplex autoimmune assay.

## Results (case reports)

Demographic and clinical details of the four patients are summarized in Table 1.

**Table 1.** Clinical and demographic features and treatments, in four patients with extensive calcinosis

Characteristic	Patient 1	Patient 2	Patient 3	Patient 4
Current age, years	68	70	51	33
Age at diagnosis of SSc^a^, years	65	65	42	27
Sex	Male	Female	Female	Female
Disease duration before onset of calcinosis (after first non-RP manifestation)	1 year	3 years	6 years	2 years
Auto-antibody profile	Anti-Scl70 +ve Anti-Ro-52 weakly +ve	Anti-PMScl-75/100 +ve	Anti-PMScl-75/100 +ve	Anti-Scl70 +ve dsDNA +ve
Creatine phosphokinase at diagnosis, U/l	443	215	1011	923
Previous and current treatments	Mycophenolate mofetil Prednisolone Minocycline	Mycophenolate mofetil Prednisolone Intravenous sodium thiosulfate	Mycophenolate mofetil Minocycline	Mycophenolate mofetil Hydroxychloroquine Prednisolone Azathioprine Intravenous sodium thiosulfate Methotrexate Rituximab

a From first non-Raynaud’s phenomenon (non-RP) manifestation.

### Patient 1

**Figure 1.**
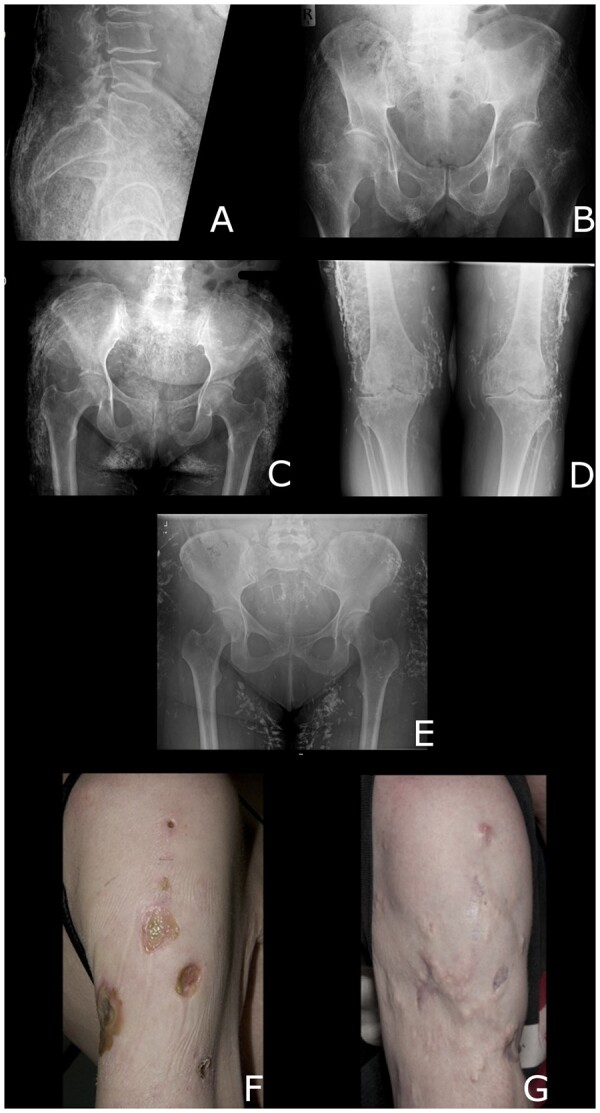
Plain radiographs (A–E) and photographic images (F, G) of calcinosis. (**A**, **B**) Calcinosis around the pelvis in patient 1. (**C**, **D**) Calcinosis around the pelvis and knees in patient 2. (**E**) Calcinosis around the pelvis in patient 3. (**F**, **G**) Areas of ulceration overlying calcinotic areas in the right upper arm of patient 4 (F) and (21 months later) the resulting disfigurement after healing (G)

This 68-year-old male was diagnosed as having diffuse cutaneous systemic sclerosis (dcSSc) and myositis overlap at the age of 65 years. His disease was characterized by Raynaud’s phenomenon (RP), skin thickening, telangiectases and myositis. Findings on clinical examination included the presence of Gottron’s papules, with some proximal muscle weakness. He had a modified Rodnan skin score (mRSS) of 23. Clinically, he had marked calcinosis around the elbows, buttocks and lateral thighs, reported onset within 1 year after his first non-Raynaud’s phenomenon (non-RP) manifestation. He was anti-Scl70 (antitopoisomerase) positive. Creatine kinase (CK) level was 443 U/l (reference range 40–320 U/l) at presentation. Plain radiography showed extensive calcinosis occurring in sheets around the pelvis (Fig. 1A, B). There were further deposits around the elbows and upper femora. Treatments used over the course of his disease included mycophenolate mofetil (MMF) and prednisolone. A 7-month trial of minocycline did not improve the calcinosis.

### Patient 2

This 70-year-old female was diagnosed 5 years previously as having an SSc-spectrum disorder with myositis overlap. Her disease was characterized by widespread skin thickening, RP, pulmonary fibrosis and marked calcinosis. Her mRSS was 14. She had lumps of calcification in her right buttock and both lower limbs posteriorly, both above and below the knees. She had developed clinically and radiographically evident calcinosis within 3 years of her first non-RP manifestation of SSc, with marked calcinosis around the hands, forearms, elbows, pelvis and thighs, well visualized on plain radiography (Fig. 1C, D). She had a CK of 215 U/l at presentation. Her autoantibody profile was positive for anti-PMScl-100 and weakly positive for anti-PMScl-75. Treatments tried included MMF and prednisolone. She had 12 intravenous (i.v.) infusions (two courses of six infusions, each administered at a dose of 25 g weekly) of the calcium chelating agent sodium thiosulphate, without any marked clinical improvement.

### Patient 3

This 51-year-old female was diagnosed with dcSSc with myositis overlap at the age of 42 years, characterized by skin thickening, RP, non-specific interstitial pneumonitis and pharyngeal involvement. On examination, mRSS was 27. Calcinosis was not evident on initial clinical assessment but became more apparent over time. Pelvic X-ray showed extensive calcinosis (Fig. 1E), with onset 6 years after first the non-RP manifestation. CK was 1011 U/l at diagnosis. Her autoantibody profile showed that she was positive for anti-PMScl-75/100. There was evidence of calcinosis on magnetic resonance imaging (MRI) of the thighs. She was treated with MMF and minocycline, with no significant change in the calcinosis.

### Patient 4

This 33-year-old female patient was 27 years old when she was diagnosed with dcSSc. Her disease was characterized by RP, overlapping inflammatory myositis, inflammatory arthritis and SLE. She developed calcinosis within 2 years of her first non-RP manifestation of SSc.

On examination, mRSS was 15. There were extensive areas of calcinosis, which discharged through the skin at multiple sites, leaving numerous ulcerated lesions of the limbs and trunk (Fig. 1F). These resulted in significant scarring and disfigurement (Fig. 1G). Her autoantibody profile included homogeneous ANA at a titre of 1/1000, positive dsDNA, low complement levels and positive anti-Scl70. CK was 923 U/l at presentation. The soft tissue calcinosis was evident on plain radiographs of the left wrist and humeri, extending along the chest wall, with further calcified deposits in the thighs and legs. MRI of her lower limbs showed generalized s.c. oedema, with diffuse non-specific skin thickening, fascial oedema and patchy muscle oedema.

Several disease-modifying therapeutic agents were tried, including MMF, azathioprine (AZA) and methotrexate (MTX). She was treated with i.v. sodium thiosulfate, of which she received a total of 12 infusions (25 g weekly), and although there was no marked improvement, it was felt that there was a possible slowing of progression of the calcinosis. Rituximab (two infusions 6 months apart) was administered for treatment of the overlapping elements of active myositis, inflammatory arthritis and SLE. This had no notable effect on the calcinosis. She required repeated courses of antibiotics for management of superimposed infections and surgical intervention for incision and drainage of the infected lesions, but the calcinosis continued to progress.

## Discussion

Widespread calcinosis of the sheet-like distribution we describe is uncommon. A small number of case reports have described this pattern in patients with connective tissue disease, when it has occasionally been labelled ‘calcinosis universalis’ [5, 6].

The pathophysiology of SSc-related calcinosis is not well understood and might differ between nodular and linear (more diffuse) forms. Several mechanisms are thought to play a major role. Vascular injury, ischaemia and hypoxia are likely to be involved [2, 7, 8]. It has been hypothesized that changes in the extracellular matrix of the skin in patients with SSc prime the environment for calcification of the soft tissues [9]. Chronic tissue inflammation and dysregulation of bone matrix proteins are also thought to contribute to the development of calcinosis [10]. Further studies are needed to provide greater insight into the pathological processes that trigger the development of calcinosis, to allow development of targeted treatments.

A well-recognized and consistent association of calcinosis is disease duration. Calcinosis usually develops after a protracted disease course, often >7.5 years after diagnosis of SSc [11, 12]. One striking point from our case series is that the four patients described all developed calcinosis within 6 years after diagnosis of SSc (mean onset 3 years after their first non-RP manifestation), consistent with the suggestion that the pathogenesis of extensive, linear calcinosis might differ from that of the commoner, nodular form.

Certain autoantibodies are associated with distinct clinical phenotypes in SSc. Traditionally, calcinosis has been believed to be more prevalent in limited cutaneous systemic sclerosis (lcSSc), previously termed CREST (calcinosis, RP, oesophageal dysmotility, sclerodactyly, telangiectasia), and often associated with the presence of ACAs [12]. However, calcinosis burden can also be high in patients with dcSSc. Other described autoantibody associations include anti-PM-Scl antibody, which is often seen in patients with overlapping myositis and SSc [13], present in two of our series of four patients.

The diagnosis of calcinosis can usually be made clinically, but several imaging modalities can be used to detect or confirm calcinosis. Plain radiographs are often sufficient as first-line imaging and well demonstrated the extent of calcinosis in all four patients described. Plain radiographs have a high sensitivity and specificity and are as sensitive as computed tomography (CT) scans in detecting calcinosis [14, 15]. On plain radiography, calcinosis can manifest as sheetlike plaques or nodules within the skin, s.c. tissues, muscle and fascia or as amorphous, cloud-like deposits [16]. CT and MRI can be helpful in providing more detail of the surrounding structures. Ultrasound (US) scans are also useful in detecting calcinosis, and some studies have suggested a sensitivity of ≤89% by US alone compared with US combined with X-ray, whilst others have reported that sensitivity of US in detecting calcinosis lesions is as high as that of plain radiography [16]. Further diagnostic techniques include PET (positron emission tomography), although this might be less favourable owing to costs and the amount of radiation exposure.

To date, there is no licensed medication for the treatment of calcinosis. Management remains challenging and is often based on anecdotal evidence, including experience from individual case reports. A paucity of randomized control trials has hampered the identification of effective therapies to manage this debilitating condition. Randomized control trials are very difficult to mount for such a rare and heterogeneous indication. At present, prompt treatment of superadded infection remains the mainstay of management. Surgical excision to debulk calcified deposits can be useful in some cases and help reduce pain and disability, although the calcinosis can regrow [17], and it was not an option in the four cases described, given the extensive nature of the calcinosis.

Several pharmacological and non-pharmacological therapies have been tried, but none is of proven efficacy. These include calcium channel blockers, bisphosphonates, i.v., intralesional or topical sodium thiosulfate, warfarin, minocycline and intravenous immunoglobulin (IVIG). Although rituximab has been proposed [18], variable results have been reported, and two infusions did not seem to improve the calcinosis in patient 4.

Topical chelator treatment is an attractive option, given that calcinosis is usually s.c. and that a locally applied (as opposed to a systemic) treatment would be less likely to cause adverse effects. Intralesional and topical sodium thiosulfate have been reported to confer some benefit [19]. However, topical chelator therapy would not be appropriate for the very widespread calcinosis present in the four patients described, two of whom were treated with i.v. sodium thiosulfate without demonstrable benefit.

We speculate that in the type of established diffuse calcinosis we describe, disease-modifying treatment (including with immunosuppressant therapy) is likely to be the most promising approach to therapy, as in juvenile DM-related calcinosis. There is emerging evidence that Janus kinase inhibitors might play a role in the treatment of calcinosis. Three patients who received tofacitinib for refractory DM and calcinosis as part of an open-label clinical trial, STIR (Study of Tofacitinib in Refractory Dermatomyositis), showed improvement in their calcinosis that coincided with improvement on muscle MRI after 3 months of treatment [20]. Janus kinase inhibitors warrant further evaluation.

In conclusion, the four cases described should raise awareness of this unusual and extensive pattern of SSc-related calcinosis, which is different from the nodular calcinosis that has been well described in the literature. Our experience suggests that when this form of calcinosis does occur, this is in the context of dcSSc and myositis overlap. Awareness of this widespread pattern of disease highlights the need for further research into the pathophysiology of this rare phenotype of debilitating calcinosis, to identify effective targeted therapies.

## Data Availability

All relevant data underlying this article are available in the manuscript. Further deidentified data cannot be shared publicly for the privacy of individuals in the case series. The data will be shared on reasonable request to the corresponding author.
